# Comparison of bioimpedance spectroscopy and X-Ray micro-computed tomography for total fat volume measurement in mice

**DOI:** 10.1371/journal.pone.0183523

**Published:** 2017-08-17

**Authors:** Gaelle Aubertin, Amira Sayeh, Jean-Philippe Dillenseger, Estelle Ayme-Dietrich, Philippe Choquet, Nathalie Niederhoffer

**Affiliations:** 1 Laboratoire de Neurobiologie et Pharmacologie Cardiovasculaire (EA7296), Faculté de Médecine, Fédération de Médecine Translationnelle, Université de Strasbourg, Strasbourg, France; 2 Preclinical Imaging Lab, UF 6237, Pôle d’imagerie, Hôpitaux Universitaires de Strasbourg, ICube, Fédération de Médecine Translationnelle de Strasbourg, Strasbourg, France; INIA, SPAIN

## Abstract

Obesity and the metabolic syndrome are two pathologies whose prevalence are in a constant increase. Evaluation of the total fat mass but also of the distribution between visceral and subcutaneous adipose tissue are important factors while assessing the pathophysiology of these two pathologies. Computed tomography (CT) and bioimpedance (BIS) are the translational methods the most frequently used in human beings as well as in rodent models in longitudinal studies on adiposity and obesity. Surprisingly, no direct comparison of micro-CT and BIS was reported yet in mice. Therefore, the present study was carried out to evaluate and compare the accuracy and the uncertainty of measurement of micro-CT and BIS in this species.

The proportion of fat mass was measured with BIS, micro-CT and direct post-mortem tissue weight, and correlations between the data were established to evaluate the accuracy of the methods but also the uncertainty of BIS and micro-CT.

There were significant correlations between weights of fat tissues on scale and proportion of total fat mass determined by BIS or micro-CT (r = 0.81 and 0.86 respectively) but both methods overestimated the total fat mass, especially in the smallest animals; overestimation of fat mass was amplified with BIS compared to micro-CT. In addition BIS and micro-CT were highly correlated (r = 0.94). Test-test reliability showed a greater variability of the BIS with respect to the micro-CT (coefficient of variation = 17.2 vs 5.6% respectively). Hence, as far as subtle differences between groups or changes within one group are awaited, micro-CT may appear as the most reliable method for determination of fat mass in mice. Micro-CT, unlike BIS, will also allow to qualitatively and quantitatively differentiate between subcutaneous and visceral adipose tissues, which is of major importance in studies on adiposity and its complications.

## Introduction

Since its first description by Reaven in 1988 [1] as “Syndrome X”, prevalence of the metabolic syndrome is in constant increase all over the world [2–7]. Between 1998 and 2009, several definitions of the metabolic syndrome have been proposed by different expert groups [8–12], all of them holding abdominal obesity as one of the diagnosis criterion. In its new worldwide definition of metabolic syndrome, the International Diabetes Federation [11] even retained central obesity as a mandatory criterion for diagnosis. Indeed, obesity is associated with a high risk of death by cardiovascular disease [13,14]. However, several studies have suggested that changes in regional distribution of adipose tissues rather than obesity represent risk factors for cardiovascular and metabolic disorders. Hence, obese patients display worse metabolic profile when abdominal obesity is associated with an excess of visceral adipose tissue than when it is associated with an excess of subcutaneous fat tissue [15–19]. These observations led Haffner [20] and Despres *et al* [21] to highlight the importance of being able to evaluate the amount of total adipose tissue and especially visceral adipose tissue, more than obesity in general.

In humans, the simplest and fastest methods to estimate the presence or absence of obesity are (i) the calculation of body mass index (BMI), defined as the ratio of weight (kg) to height (m^2^) and (ii) the measurement of waist circumference (cm). If the BMI allows to classify the population in underweight (BMI<18,5 kg/m^2^), normal (18,5≤BMI<25 kg/m^2^), overweight (25≤BMI<30 kg/m^2^) or obese (≥30 kg/m^2^), the relationship between BMI and the proportion of body fat depends largely on age, gender and ethnicity [22]. In the same way, Despres *et al* [21] reported that waist circumference is a better predictor of visceral adipose tissue than BMI. For a long time, the gold standards for the quantification of total adipose tissue and especially visceral adipose tissue have been X-Ray computed tomography (CT) and magnetic resonance imaging (MRI) [22]. However, these two imaging methods are expensive and need specialized staff and specific installations; CT also exposes patients to ionizing radiations. Due to these disadvantages, new methods based on impedance measures were developed, *i*.*e*., single or multiple frequency bioimpedance analysis (BIA) and bioimpedance spectroscopy (BIS). These methods are non-invasive, relatively inexpensive, portable and do not require extensive operator training; therefore, they are increasingly used to assess body composition in many clinical applications, as prognostic indicator of disease severity, morbidity, hydration status, malnutrition or lymphedema [23,24]. However, if conventional BIS gives the proportion of adipose tissue, it does not provide information on the distribution of adipose tissue within the body. Recently, a new dual-BIA instrument has been developed to assess intra-abdominal fat area and subcutaneous fat area, by measuring truncal impedance and surface impedance at the abdomen [25].

As stated above, obesity seems to play a crucial role in the development of metabolic syndrome, but despite intensive research, the pathophysiological mechanisms involved remain poorly understood and efficient anti-obesity treatments are still lacking. Rodent animal models are the most largely used in preclinical studies to identify pathophysiological mechanisms and new therapeutical strategies for obesity, type 2 diabetes mellitus, dyslipidemia or metabolic syndrome. The determination of adipose tissue proportion and distribution in these animals is therefore very important. Furthermore, since these pathologies usually evolve over several months, methods allowing longitudinal follow-up of the animals are needed.

The first method to determine the animal total fat mass (FM) is to recover and weigh all adipose tissues after sacrifice. Logically, this method does not allow to follow the evolution of the fat mass in the same animal over time. Another way to reach the proportion of fat mass is by using isotopic dilution. Animals are injected deuterium-labeled water (D_2_O) and/or H_2_^18^O; the monitoring of the kinetics of deuterium (and/or ^18^O) equilibration and disappearance allows the calculation of the total body water (TBW), then the deduction of the fat-free mass (FFM) and the FM [26,27]. This method has been used in many animal species, including rats, but since it requires repeated blood sampling, it is hardly applicable to the longitudinal follow-up of mice. Moreover, as far as translational studies are concerned, it may appear more appropriate to use the same method of body composition analysis in animals and humans. Dual X-ray absorptiometry (DEXA) and quantitative nuclear magnetic resonance (qNMR) have also been used successfully to quantify the FFM and FM, but they do not provide spatial distribution of adipose tissue [28–30]. X-Ray micro-computed tomography (μCT) is a well-established method since about ten years to determine adipose tissue proportion in rodents [31–36]. Advantages of μCT are multiple: it is non-invasive and relatively quick, enables repeated measurements over time in the same animal and provides detailed spatial information on body fat distribution. Limits are the image processing (manual or semi-automatic fat segmentation), which is time consuming, and exposure to ionizing radiation. Finally, over the last decade, the use of BIS to determine the body composition in small animals has spread from rat models [37–40] to mouse models [41]. Similarly to μCT, this method is rapid; additional advantages are the low cost and the possibility to obtain at once the proportion of the different body compartments (TBW, FFM and FM). However, the procedure is more invasive than μCT due to the placement of needles and devices designed for BIS measurements in small animals do not allow to differentiate the various adipose tissues.

Accuracy of μCT and BIS in rodents have been validated by comparing values of body composition obtained with reference techniques. Hence, quantification of adiposity by μCT was mostly compared to direct post-mortem weighing of abdominal and subcutaneous fat pads [31,33–35] and more recently, to in vivo qNMR in mice [30]; body composition evaluated by BIS was correlated to values obtained by isotopic dilution and chemical carcass analysis [38,39,42]. To our knowledge, there is however no report of a comparison between μCT and BIS in mice. Therefore, the present study was carried out to evaluate and compare the accuracy and the uncertainty of measurement of μCT and BIS in this species. For this purpose, the proportion of adipose tissue was measured with these two methods and by direct post-mortem tissue weigh, and correlations between the data were established. Intra-mouse variations were also calculated. These data may help identifying the most adapted method according to the pursued objectives, and thus making the choice between μCT and BIS when designing a new study in mice.

## Materials and methods

### Animals

A total of 51 male C57Bl/6J mice were housed in standard conditions (12 hours light/dark cycle, temperature controlled) and had *ad libitum* access to food and water. A first group of 5 mice was used to determine the density of visceral and subcutaneous adipose tissue (see below). A second group comprising 24 mice was defined, in which fat mass (FM) was evaluated by BIS, μCT and in some of them, adipose tissue weight (see below); data served for the evaluation of uncertainty of the BIS and μCT methods (Study 1, n = 3) and for the analysis of the correlations between the different methods of measurement of FM (Study 2, n = 21). A third group of 10 mice was used to evaluate the inherent error of the *in vivo* measurement (due to physiological movements) by the μCT (Study 3). Finally, a fourth group of 12 mice (aged of 25 weeks) was used to validate the use of μCT for the assessment of FM increase in a longitudinal study (Study 4). Mice were randomized to receive either normal diet (n = 6; A04 diet, SAFE, Augy, France) or enriched diet (n = 6; high fat high glucose diet containing 12.5% added bacon, 0.4% added cholesterol and 33% added glucose, SDS Dietex, Argenteuil, France) for 15 weeks.

All procedures were conducted in accordance with the French and European regulations on experimentation in mammals. Experiments were approved by the animal ethics committee of the Strasbourg University.

### Determination of fat density

**Fig 1:**
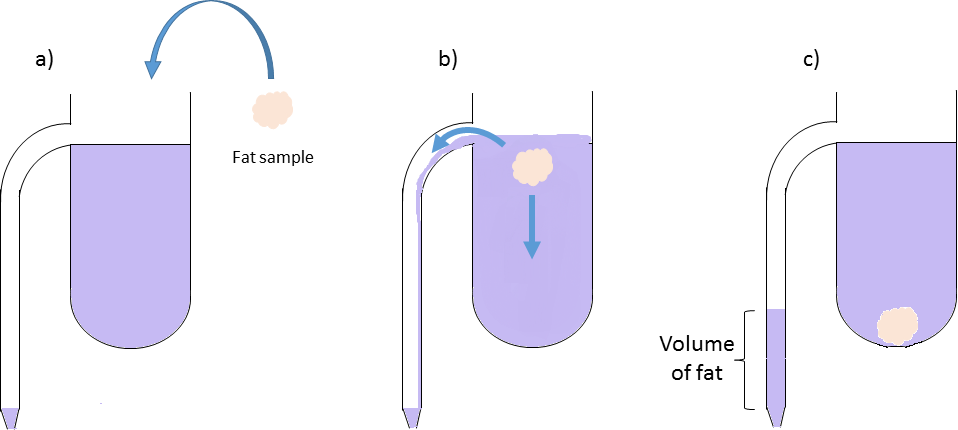
Scheme of the use of the density meter. The density meter comprises a graduated tube connected to a tank, filled with an inert liquid (kerosene) (43,44). (a) A sample of mouse fat is weighed and placed slowly in the tank. (b) The fat sample overflows the kerosene that falls into the graduated tube. (c) The volume of kerosene (mL ± 0.05mL) that has flowed into the graduated tube is measured and is equal to the volume of fat.

Difficulty to find precise references for the value commonly used pushed us to develop a method of measurement of fat density. Based on the work of Ali *et al* [43], we designed an overflowing density meter (**Fig 1**, Verlabo 2000, Strasbourg, France). As the density of fat is lower than water, kerosene was used as inert liquid to measure the volume of fat [44]. Animals were anesthetized with pentobarbital (60 mg/kg intraperitoneally, Pentobarbital^®^ 6%, Ceva, France), then sacrificed with an overdose of pentobarbital. Visceral and subcutaneous adipose tissue samples were immediately harvested. For the determination of density, a piece of fat was weighed and placed slowly in the density meter tank; the volume of kerosene (mL ± 0.05 mL) that had flowed into the graduated tube was then measured. Five consecutive measurements were performed for a single fat sample (weight and corresponding volume). The density of fat was calculated by applying the standard formula, $d=\frac{m}{v}$ given in mg/mL. This protocol was repeated for a total of 10 samples (5 abdominal and 5 subcutaneous adipose tissue).

### Determination of fat mass

All measurements were carried out by the same experimenter and in the same conditions.

#### Measurement of fat mass by bioimpedance spectroscopy (BIS)

**Fig 2:**
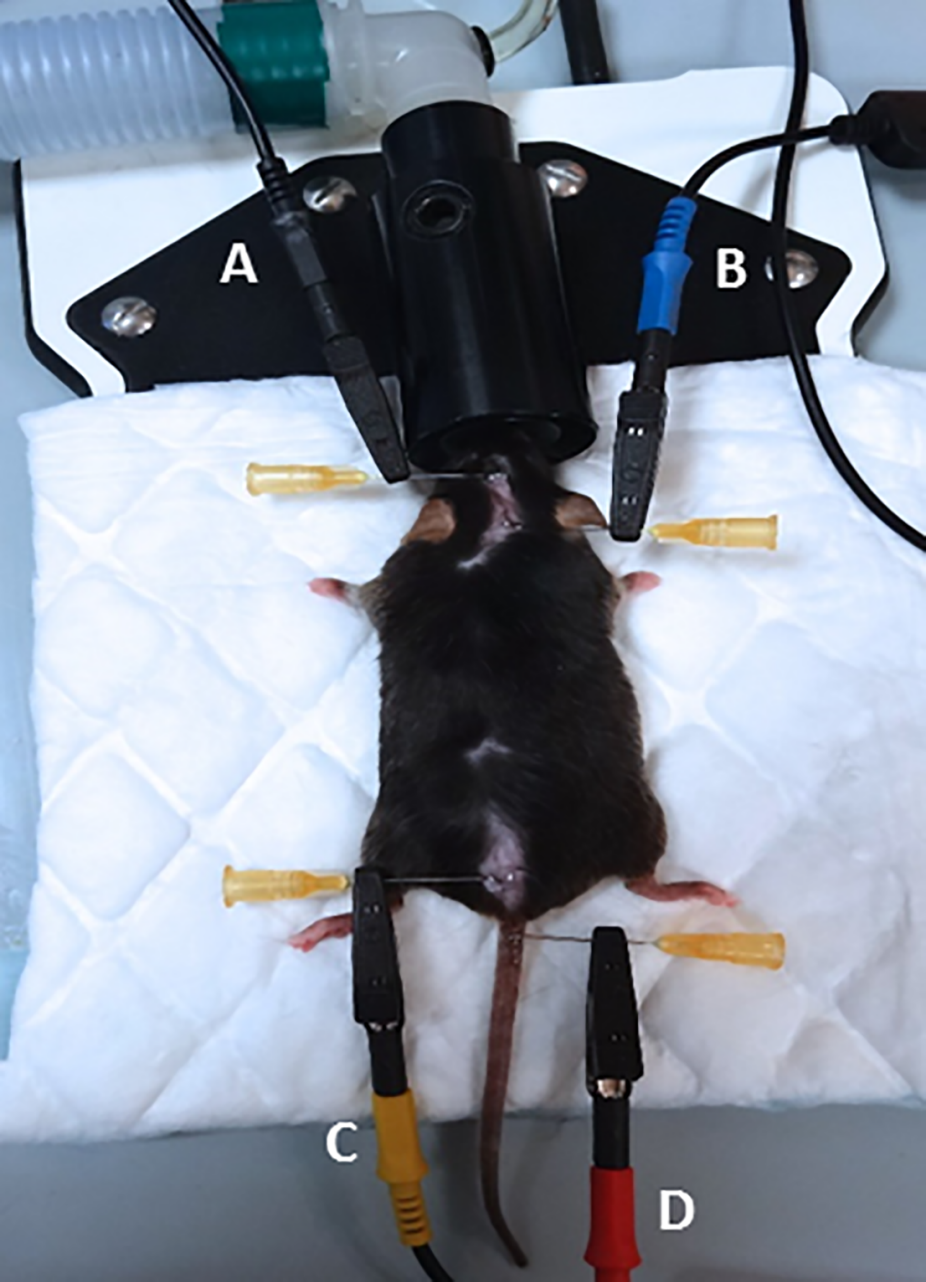
Placement of bioimpedance spectroscopy electrodes on a mouse. Four 25G needles are placed subcutaneously along the midline of the back and serve to connect the electrodes to the animal. The B electrode (blue) is placed at the intersection between the median line and the line between the ears. The A electrode (black) is placed 1 cm from B towards the muzzle. The C electrode (yellow) is placed between the median line and that joining the thigh muscles. The D electrode (red) is placed 1 cm backwards at the base of the tail. The length between B and C needle electrodes is measured; this parameter, as well as the age and weight of the animal, is required by the software to calculate the proportion of fat mass.

The whole body composition was measured with the ImpediVet Bioimpedance Spectroscopy device (ImpediMed Limited, Brisbane, Australia) as described previously [38]. Mice were weighed, anesthetized with 2% isoflurane (Iso-Vet, Piramal Healthcare UK Limited, United Kingdom) pushed by air and placed in prone position on a temperature-controlled table (Minerve, Esternay, France) with a non-conductive single-use absorbent support. Hair from the head and the back were removed for placement of electrodes. Body position was standardized: fore- and hind-limbs on each side of the body and tail extended distally. Four 25G needles, used as electrodes, were placed subcutaneously along the midline of the back according to manufacturer’s instructions (**Fig 2**). The length between “B” and “C” electrodes was measured; this length, together with age and body mass of the animal is used for the calculation of the different parameters given by the BIS device. Thirty continuous BIS measurement (1 measurement/s) were acquired from 4 kHz to 1MHz. The acquired data were downloaded and analyzed with the proprietary software (ImpediVet BISI version 1.0.0.4) to determine TBW (L) and global FM (%). The whole procedure took less than 10 minutes.

#### Measurement of fat mass by micro-computed tomography (μCT)

Following anesthesia with isoflurane (1.5–2%) pushed by air, mice were laid in a supine position in an imaging cell (Minerve, Esternay, France), in which the animal was kept anesthetized, warmed and could be monitored (respiration and/or ECG, Minerve Esternay, France). Three-dimensional x-ray images were acquired on the CT part of a μSPECT-μCT (eXplore speCZT Vision 120, General Electric, Waukesha, USA). Three fields of view were necessary to acquire whole body and the total acquisition time was 15 min. The tube voltage was set at 80kV with a constant 32mA current. All acquired volumes were reconstructed with a voxel size equal to 100x100x100 μm^3^. Reconstructed and stitched together images to obtain a whole body volume, were filtered with a Gaussian filter to reduce noise.

**Fig 3:**
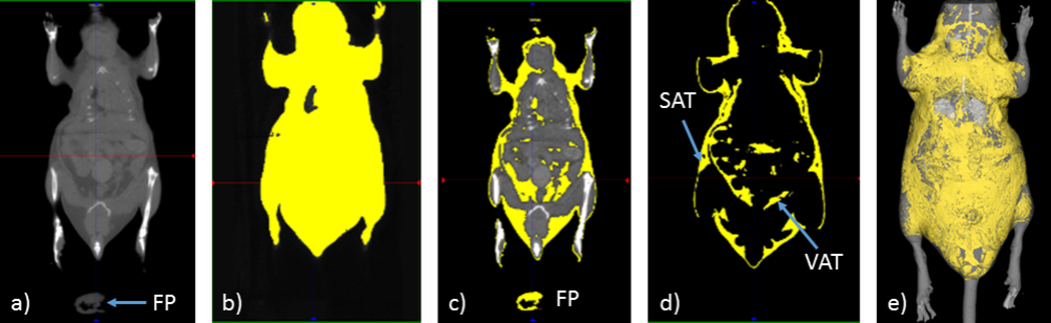
The different steps for quantification of total adipose tissue by micro-computed tomography. Anesthetized mice are placed in a supine position in an imaging cell; three-dimensional x-ray images are acquired on the CT part of a μSPECT-μCT (eXplore speCZT Vision 120, GE, Waukesha, USA). Volumes are reconstructed with a voxel size equal to 100x100x100 μm^3^; reconstructed images are filtered with a Gaussian filter to reduce noise. (a) Whole body scan. (b) Selection of whole body volume. (c) Choice of threshold level based on fat pad (FP). (d) Representation of total adipose tissue with subcutaneous adipose tissue (SAT) and visceral adipose tissue (VAT). (e) Isosurface representation of whole body in grey and fat in yellow.

Because X-ray are less attenuated by fat than by soft tissues, it could be isolated from the other structures (air, bones and soft tissues) by choosing thresholds based on the Hounsfield unit values. Values for the thresholds were established using a fat pad placed behind the animal, which was also taken in the field of view (see **Fig 3A**). The volume of the fat pad was calculated from the sample weight and fat density (see above). On the reconstructed images, threshold was set so that the volume of the fat pad determined from density matched the calculated volume (**see Fig 3C**) and therefore makes it possible to be independent from the operator. For determination of whole body volume, thresholds ranged from -350 to +3000 Hounsfield units (HU); for fat volume segmentation, thresholds ranged from -350 to -150 HU. The proportion of global FM (%) was calculated as the ratio between the volume of total (subcutaneous and visceral) adipose tissue (mm^3^; see **Fig 3D**) and the whole body volume (mm^3^; see **Fig 3B**).

#### Measurement of fat mass by autopsy

At the end of the different procedures, anesthetized mice were sacrificed with a lethal dose of pentobarbital (120mg/kg, intraperitoneally). Adipose tissues (visceral and subcutaneous) were harvested and weighed on a precision scale (Mettler Toledo PL200, ± 0.001g, Zürich, Switzerland).

### Study design

#### Study 1: Test-test reliability

To assess the uncertainty of BIS and μCT methods, body composition measurements were performed with the two methods on 3 mice, three times a day (morning, noon and evening), during five consecutive days. For each method and each animal, coefficients of variation (CV, see below) were computed between all measurements (n = 15 values/animal). A second comparison is related to the day, in this case mean was calculated for each day (n = 5 values/animal). A third comparison is related to the time (morning, noon and afternoon), in this case mean was calculated for each time on the five days (n = 3 values/animal). For all animals, anesthesia lasted less than 30min and recovery from anesthesia occurred within 5-10min; mice were placed in their own cage and had full access to food and water between the different sessions.

#### Study 2: Comparison of total body fat mass determined by BIS, μCT and autopsy

Twenty one mice of variable body weight (26 to 36g) were weighed, then anesthetized with isoflurane to measure the proportion of FM by BIS, as described above. Immediately afterwards, and without awakening, each animal was subjected to μCT scan. Correlations were calculated between total body weight and fat estimation by BIS or μCT, and between FM values derived from BIS and μCT. Autopsy took place within 24 hours after the measurements by BIS and μCT. Total adipose tissue was recovered and weighed; correlations between the weight of harvested adipose tissue and the FM obtained by BIS or μCT were calculated.

#### Study 3: Comparison of ungated and gated scans

**Fig 4:**
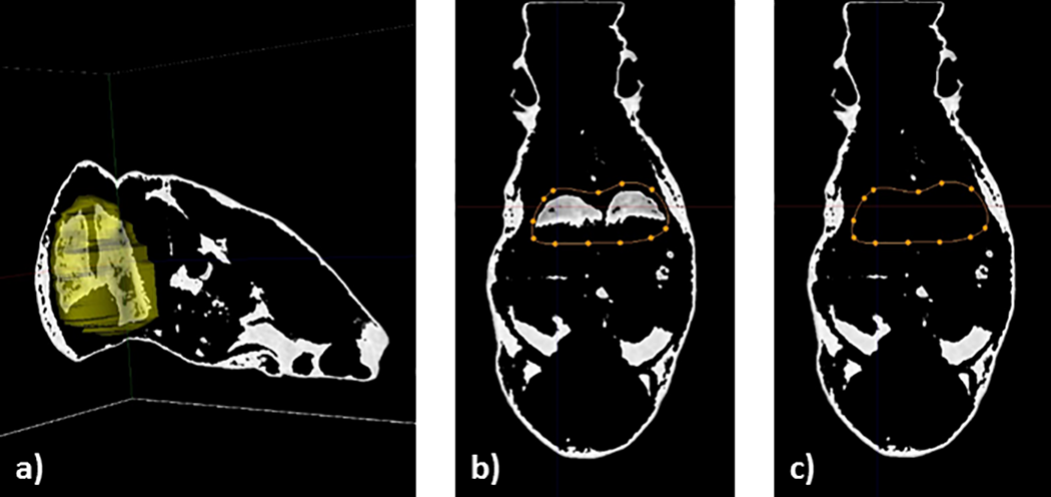
The different steps for lung manual correction on fat volumes on micro-computed tomography scans. Micro-CT scans were performed in 10 mice, with and without respiratory-gated conditions, on the same field of view (thoracic region). (a) 3D representation of the region of interest around the lungs. (b) One slice of representation of total adipose tissue with lung selection through a region of interest (in orange dotted line). (c) Suppression of lung region on the same slice.

Due to physiological, i.e., respiratory movements of the animals, partial volume effect is noticeable at the edges between air and soft tissues, especially at diaphragmatic cupola. Hence the CT value of the voxels at these edges decrease and so these voxels might be considered as adipose tissue. This is especially true for lungs and may require manual correction. These artefacts may be minimized by respiratory-gated acquisitions. However, gated-scans increase scanning-time. In order to test whether results differ between scans performed in respiratory ungated animals and gated animals, we studied fat distribution in thoracic region of 10 mice and repeated the scans with respiratory-gating on the same field of view using the same threshold values. The scans were then analyzed without and with manual correction (*i*.*e*. suppression) in lungs area (**Fig 4**).

#### Study 4: Validation of μCT for the follow-up of fat mass increase in longitudinal study

Twelve male mice were randomized into 2 groups (n = 6 each) received normal (ND) or high fat diet (HFD) for 15 weeks. All animals had free access to food and water. Animals were weighed and submitted to *in vivo* μCT scans as described above, before and after 15 weeks of diet. All measurements were performed at the same time of day. The proportion of global FM (%) was determined for each animal at each time and the mean differences between the beginning and the end of the diet were compared.

### Statistical analysis

Results are given as mean ± SD. Correlations between the different parameters were studied using linear regression analysis. Uncertainty of BIS and μCT measurements are presented as coefficients of variation (CV), defined as the standard deviation (SD) divided by the mean of the measures and multiplied by hundred. In Study 4, changes in body weight and FM were analyzed by 2-way analysis of variance (with diet and time as factors) (GraphPad Prism 6.0 software, La Jolla, United States of America). The significance level of the results was set at α = 0.05.

## Results

### Determination of fat density

For all the different measurements, the mean fat density was 0.92 ± 0.01 mg/mL. There were no significant differences between abdominal fat density and subcutaneous fat density (0.92 vs 0.92 mg/mL respectively, p = 0.999).

### Study 1: Test-test reliability

The results for test-test reliability are presented in **Table 1**. The CV for all the measurements of total adipose tissue (3 mice, 15 values/mouse at different times and days) ranged between 15.3% and 20.3% for BIS and between 3.5% and 7.4% for μCT.

**Table 1:** Coefficients of variation of total adipose tissue measurements by bioimpedance spectroscopy and micro-computed tomography.

	Coefficients of variation (%)		
	All measurements (n = 15 values/animal)	Over time at the same day (n = 5 values/animal)	Over day at the same time (n = 3 values/animal)
**BIS**	17.2 ± 2.7	13.6 ± 7.0	3.5 ± 2.2
**μCT**	5.6 ± 1.9 *	4.1 ± 3.0	1.2 ± 0.3

The variation of the measure, after repositioning of the animal during the same day, is weaker with μCT than with BIS (4.1±3.0% vs 13.6±7.0% respectively, p = 0.10). Similarly, when comparing the variation of the two methods while measuring at the same time (morning, noon or evening) along 5 days, the CV was three times lower for μCT than for BIS (1.2±0.3% vs 3.5±2.2% respectively, p = 0.15).

Body composition measurements were performed with bioimpedance spectroscopy (BIS) and micro-computed tomography (μCT) methods on 3 mice, three times a day (morning, noon and evening), during five consecutive days. For each method and each animal, coefficients of variation (CV) were computed between all measurements (n = 15 values/animal), between the time of measurements on the same day (n = 5 values/animal) and between the days of measurements at the same time (n = 3 values/animal). CV are defined as (standard deviation/mean) x100. Data are presented as mean of the 3 mice ± SD. Statistical analysis was performed using a one-way ANOVA analysis and a p<0.05 was considered significant. *: p<0.05 μCT *versus* BIS.

### Study 2: Comparison of total body fat determined by BIS, μCT and autopsy

**Fig 5:**
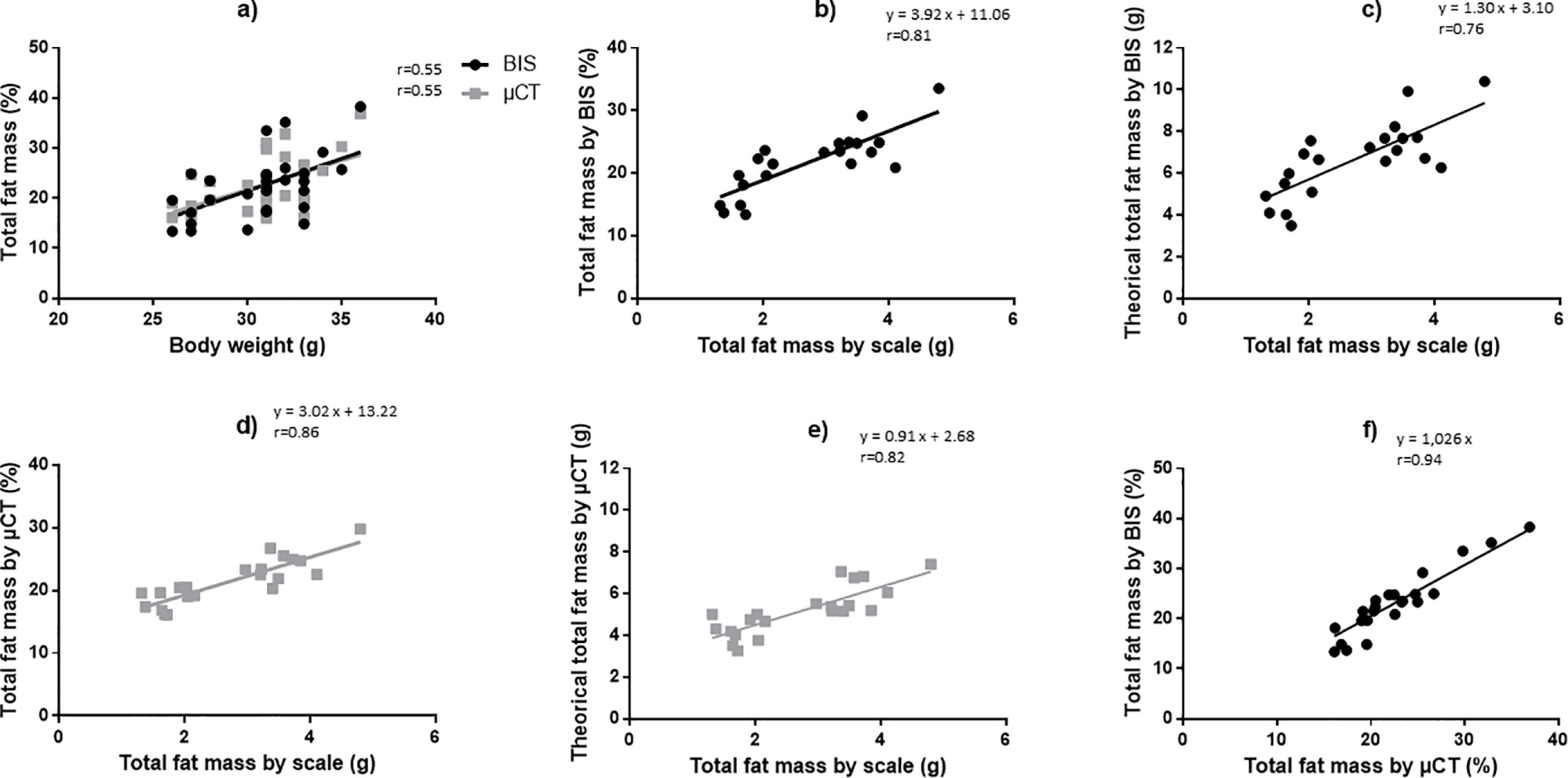
Relationships between the different methods of determination of total adipose tissue. Thirty mice (body weight ranging from 26 to 36g) were used. In each animal, the proportion of adipose tissue was measured with bioimpedance spectroscopy (BIS), micro-computed tomography (μCT) and by direct post-mortem tissue weigh as described in Material and Methods. Theoretical adipose tissue weight was calculated from body weight and BIS or μCT data. (a) Correlation between the body weight of animals and the proportion of total fat mass estimated by BIS and μCT. (b-e) Correlations between the weight of harvested adipose tissue and the proportion of fat mass estimated by BIS (b) or μCT (d) and between the weight of harvested adipose tissue and the theoretical calculated adipose tissue weight obtained by BIS (c) or μCT (e). (f) Correlation between total adipose tissue estimated by BIS and μCT. r is the Pearson correlation coefficient.

Volumes for total, fat and lean tissues determined by μCT (n = 21) were: 26.00 ± 2.44 cm^3^, 5.61 ± 1.26 cm^3^, and 18.59 ± 1.01 cm^3^, respectively, yielding a ratio of 21.44 ± 3.62% for fat/total volumes and a ratio of 68.97 ± 2.10% for lean/total volumes. For each animal, adipose tissue proportions estimated by BIS and μCT were plotted against body weight (**Fig 5A**) and against weight of harvested fat (**Fig 5B and 5D).** There was a significant correlation between the body mass (g) of the mice and the total FM (%), be it estimated by BIS or μCT (r = 0.55 and p<0.05 for both methods). The correlation coefficient between *ex vivo* fat mass (g) and the proportion of FM was slightly higher when estimated by μCT compared to BIS (r = 0.86 and 0.81 respectively; p<0.001 for both). For both methods, the calculated theoretical fat weight was significantly correlated to the weight of harvested adipose tissue (**Fig 5C and 5E**) but was always overestimated; when using μCT, the Pearson correlation coefficient was however largely higher and the overestimation was less than with BIS.

Comparing the two methods, proportions of total adipose tissue estimated by μCT and BIS were strongly correlated (r = 0.94, p<0.001; **Fig 5F**). However, the slope of regression line was >1.0 indicating that FM values assessed by BIS were slightly higher than those estimated by μCT.

### Study 3: Comparison of ungated and gated scans

There was no statistically significant difference between the proportions of adipose tissue in the thoracic region measured by μCT with or without respiratory-gating and before and after manual lungs area correction (see **Table 2**). Values ranged from 22.95 ± 4.01% to 25.04 ± 3.73%, with a maximal error of 2.1± 0.7%.

**Table 2:** Proportion of total adipose tissue in thoracic region.

	Proportion of total adipose tissue in thoracic region (mean ± SD)		
	Without manual lungs area correction	With manual lungs area correction	p
**Ungated scan**	25.04 ± 3.73%	23.36 ± 3.91%	0.34
**Gated scan**	24.14 ± 3.82%	22.95 ± 4.01%	0.51

Micro-CT scans were performed in 10 mice, with and without respiratory-gated conditions, on the same field of view (thoracic region). Scans were then analyzed with and without manual correction in lungs area. Data are presented as mean ± SD of the 10 mice. Statistical analysis was performed using a one-way ANOVA analysis.

### Study 4: Validation of μCT for the follow-up of fat mass increase in longitudinal study

**Fig 6:**
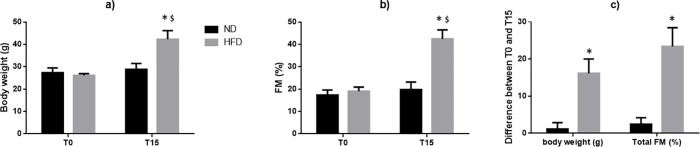
Effect of a normal or high fat diet on body weight and proportion of total fat mass estimated by micro-computed tomography in mice. Mice received normal (ND) or high fat (HFD) diet for 15 weeks. Animals were weighed and the proportion of total fat mass (FM, %) was determined by micro-computed tomography (μCT) scans as described above before (T0) and at the end (T15) of the treatment period. Evolution of (a) body weight and (b) FM over time. (c) Mean increases in body weight and total FM after 15 weeks of diet. Data are represented as the mean ± standard deviation of 6 animals in each group. Differences in body weight and FM were analyzed by 2-way analysis of variance (with diet and time as factors) (GraphPad Prism 6.0 software). *: p<0.05 HFD *versus* ND. $: p<0.05 T15 *versus* T0 within the same group.

There was no significant differences in initial body weight (27.3±2.2 g vs 26.2±0.8 g for ND and HFD groups, respectively) and in FM (17.3±2.3% vs 19.8±3.3% for ND and HFD groups, respectively) among the two groups **(Fig 6)**. After 15 weeks, mice fed with ND displayed no significant increase of body weight or FM (+1.5 g and +2.5% respectively, p>0.05 versus T0); inversely, body weight increased significantly in the HFD group (+16.2 g, p<0.05); this was associated with a significant increase in FM (+23.4%) measured by μCT **(Fig 6)**. In both groups, lean tissue volumes were similar; due to the increase in total volume in HFD mice, lean/total volume ratio was significantly reduced in these animals compared to ND mice (**Table 3**).

**Table 3:** Total, fat and lean volumes, and calculated fat/total volume and lean/total volume ratios in mice after 15 weeks of normal (ND) or high fat (HFD) diet.

	Volumes and ratio of adipose and lean tissues (mean ± SD)				
	Total volume (cm^3^)	Fat volume (cm^3^)	Lean volume (cm^3^)	Fat/total ratio	Lean/total ratio
**ND**	24.09 ± 1.98	4.75 ± 0.86	17.23 ± 1.41	0.20 ± 0.03	0.72 ± 0.03
**HFD**	38.94 ± 4.25*	16.65 ± 3.18*	19.46 ± 1.38	0.43 ± 0.04*	0.50 ± 0.04*

Mice received normal (ND) or high fat (HFD) diet for 15 weeks (n = 6 in each group). Micro-CT scans were performed at the end of the treatment as described above. Data are presented as mean ± SD. Statistical analysis was performed using a one-way ANOVA analysis.

*: p<0.05 HFD *versus* ND.

## Discussion

In this study, we compared for the first time the accuracy and uncertainty of BIS and μCT, two methods allowing the assessment of the proportion of adipose tissue in mice. Indeed, BIS and CT are the translational methods the most frequently used in human beings [45–47] as well as in rodent models [31–35,37–42] in longitudinal studies on adiposity and obesity. In clinical research, only two studies have compared these two methods [48,49]. Both reported strong correlations between the different methods. However, Park *et al* [48] showed significant overestimation of visceral adipose tissue by dual-BIA in the larger ranges of values of fat mass. Surprisingly, no direct comparison of μCT and BIS was reported yet in mice.

First, we developed a semi-automated method for the segmentation and quantification of adipose tissues by μCT. Discrimination of fat from other tissues is achieved by applying a threshold in Hounsfield units that isolates density voxels of fat tissue from the others. Consequently, reliability of fat mass measurements relies on the choice of the most appropriate threshold. In our experiments, the threshold value was determined by matching the mass of a sample of harvested fat to the volume calculated by μCT and converted into mass using 0.92 mg/mL as fat density value. This value was measured with our density meter and is the same as that used by others [50,51]. The main interest of this first work is to have developed a semi-automated method for the segmentation which is independent of the operator; of note, threshold ranges used to discriminate tissues, as well as total fat and lean volumes obtained in mice were similar to values reported previously [36]. We also ensured that the total adipose tissue volume determined using our procedure was not overestimated by the respiratory movements of the animals and the presence of air within lung tissues. Approaches to limit miscategorizing of lung air as adipose tissue are (i) to perform respiratory-gated acquisitions or (ii) to perform manual segmentation around the lung area. After analyzing our data with and without synchronization and for each case, with and without manual segmentation, we did not observe any significant differences in the measured proportion of total adipose tissue in the thoracic region. The maximal error between the different conditions was about 2%, which is within the range of variability of the μCT procedure (1.2–5.6%). These data suggest that both corrections, which are time consuming and considerably lengthen the duration of the whole μCT procedure, are not required in mice. Since duration is usually regarded as a major limit of the method, this result may represent a strong argument favoring the use of μCT in mice.

To validate the accuracy of μCT and BIS in mice and compare the two methods, total adipose tissue was evaluated by μCT or BIS and values were matched to the mass of adipose tissue resected at autopsy of the animals.

FM proportions estimated by μCT and BIS over a large range of values were strongly correlated (r = 0.94); of note, BIS values were slightly (3%) higher than μCT values and, more importantly, were more fluctuating than μCT values. Hence, for a FM measured by μCT settled at 20%, BIS displayed FM values varying from 14% to 22% (-30% to +10%); in contrast, for a FM measured by BIS settled at 20%, μCT values varied from -10% to +15% only. Total FM obtained by μCT or BIS was also highly correlated with the weight of harvested adipose tissue (r = 0.86 and r = 0.81, respectively). This is in agreement with previous studies which validated μCT measurement of adipose tissue against partially [31–33] and completely [34,35] resected adipose tissue weights or compared the mass of total adipose tissue obtained by BIS with that obtained by chemical analysis carcass [38]. In all cases, very good correlations were reported, with correlation coefficients ranging from 0.91 to 0.99. However, when calculating a theoretical fat mass from the BIS or μCT data and animals body weight, we noticed that both methods overestimated the fat mass, especially in the smallest animals, and that overestimation was amplified with BIS compared to μCT (theoretical fat mass ranging from 3.5-6g and 3.2–4.2g for BIS and μCT respectively, in mice with 1.8g of fat measured at autopsia). Similar findings were reported previously [35,38]. One possible explanation may reside in the difficulty of harvesting all the fat, particularly in the abdominal region, leading to underestimation of the total fat mass in animals. This, however, holds true for both methods and can therefore not account for the differences observed between BIS and μCT. A plausible explanation for this difference is the higher variability of the BIS measurements. While assessing the reliability of the two methods, we found coefficients of variation three times higher for BIS than for μCT. This can be explained by the fact that the value given by BIS, unlike μCT, is extremely dependent on the hydration animal status, the position of the animal and the placement of the needle electrodes [38]. In agreement, FM values varied considerably over 15 independent measurements (3x/day, 5 consecutive days), with a coefficient of variation of 17%. Our data show that the time of measurement represents the most important factor of variability (CV = 13.6±7.0% for data obtained at morning, noon and afternoon), most probably reflecting changes in the nutrition and hydration status of animal along the day. Thus, performing BIS evaluation always at the same time seems crucial to improve reliability. The influence of the time of measurements is much less pronounced for μCT. Based on our results we cannot reject the importance of measuring at the same time although for μCT. In the BIS procedure, accuracy in the determination of the distance between B and C electrodes also plays a key role. This length, together with age and body mass of the animal, is used for the calculation of the different parameters given by the device. Small differences in the measurement of this distance may produce large fluctuations in the calculated FM, accounting for high variations of the BIS method. Moreover, accuracy of the distance measurement likely decreases in small animals, which could contribute to the enhanced variability in this particular population. Finally, we noticed that intrinsic variability of BIS is much higher in animals with low FM: coefficients of variation within one session (30 continuous measurements during 30 seconds) were 8.6 to 19% in animals with low FM (13–15%) versus 2.7 to 6.7% in animals with high FM (35–38%). Although not fully understood, this observation may also partly explain why BIS overestimation was enhanced in small animals. In their studies using μCT, Hillebrand *et al* [34] and Lubura *et al* [35] noticed CV values of the same range than ours (4.5 and 2.2% vs 1.2 to 5.6%, respectively). Looking at BIS variability, a day-to-day variation of 3.5% has been reported [39], in accordance with the CV obtained in the present study (3.5±2.2). In contrast, Smith *et al* [38] found a CV of 5.4% on 2 successive sessions performed on the same day, after repositioning the needle electrodes, compared to our value of 13.6% on 3 successive scans. This large discrepancy may be explained by the fact that in their study, animals were not awakened between the two successive sessions, while in our study, animals were allowed to recover from anesthesia during several hours and were placed in their own cage with full access to food and water before repeating the measurement.

Finally, animal body weight and FM estimated by BIS and μCT were significantly correlated. However, the calculated coefficients of correlation were relatively low (r = 0.55 for both methods) and, for a same given body weight, the proportions of total fat mass varied greatly (even from single to double in some cases), irrespective of the method used. These data confirm that body weight *per se* is not a good prognostic factor for the presence of obesity [52–54]. In order to validate μCT as a valuable tool for the long-term follow-up of mice, we designed a study in which animals were proned to develop obesity through enriched diet. In all animals, the proportion of FM was evaluated on one day at the same time, *i*.*e*., in conditions which were set as the most reliable (CV = 1.2±0.3%). As expected in adult mice under normal diet, the proportion of FM increased only moderately over the 15 weeks (+2.5%), but the difference was beyond the detection threshold of the μCT. In contrast, enriched diet induced a 2-fold increase in total FM. These data confirm that μCT represents a precise and suitable tool for detecting moderate as well as large increases in FM in mice over a long period of time. In addition and in agreement with our conclusion, Upadhyay *et al* [55] demonstrated that μCT is also valuable to highlight small (<10%) reductions in the amount of white adipose tissue induced by pharmacological treatments.

In summary, our data show that BIS and μCT are two valid methods to assess the proportion of fat mass in mouse models of obesity or metabolic syndrome. However, BIS values display a much greater variability, limiting its sensitivity. Hence, as far as subtle (*i*.*e*., < 5–10%) differences between groups or changes within one group are awaited, μCT may appear as the most reliable method for determination of FM. μCT, unlike BIS, will also allow to qualitatively and quantitatively differentiate between subcutaneous and visceral adipose tissues, which is of major importance in studies on adiposity and its complications.
